# Industrial Perspective on the Manufacturing of Lipid Nanoparticles for Nucleic Acid Delivery

**DOI:** 10.3390/pharmaceutics18040489

**Published:** 2026-04-16

**Authors:** Jenny Hong Hoang, Melanie Ott, Eleni Samaridou, Moritz Beck-Broichsitter, Johanna Simon

**Affiliations:** 1 Merck Life Science KGaA, 64293 Darmstadt, Germany; jenny.hong-hoang@merckgroup.com (J.H.H.); ott.melanie@gmx.de (M.O.); eleni.samaridou@merckgroup.com (E.S.); moritz.beck-broichsitter@merckgroup.com (M.B.-B.); 2 Institute of Pharmacy and Molecular Biotechnology, Heidelberg University, 69117 Heidelberg, Germany

**Keywords:** lipid nanoparticles (LNPs), nucleic acid delivery, gene therapy, manufacturing scale-up, mixing technologies, critical quality attributes (CQAs), tangential flow filtration (TFF)

## Abstract

Lipid nanoparticles (LNPs) have emerged as a groundbreaking delivery platform, revolutionizing the development of nucleic acid-based medicines for gene delivery and gene therapy. This review provides an insightful industrial perspective on the production process of LNPs, focusing on cutting-edge manufacturing equipment, downstream processing and the crucial transition from laboratory to large scale. While LNP production in the discovery phase relies on a small scale (µL to mL) for screening various LNP formulation candidates, transferring to preclinical (up to hundreds of mL) and clinical/commercial scales (up to liters) requires a robust and reproducible manufacturing process. Thus, mixing technologies throughout these scales must be carefully selected and require precision, scalability and high reproducibility to meet the target quality of the LNP drug product. Key mixing technologies in mRNA-LNP production primarily include microfluidic systems and impinging jet mixers (IJMs). In this review, we discuss key critical process parameters (CPPs) in LNP preparation, including flow rate ratio (FRR) or total flow rate (TFR), in relation to associated critical quality attributes (CQAs) across multiple manufacturing scales. We further assess the impact of downstream processing, specifically tangential flow filtration (TFF), on the formulation’s CQAs. In particular, the review highlights the importance of maintaining CQAs along each step of the process and emphasizes the role of robust analytical methods in ensuring product quality and safety. Additionally, we touch on current challenges associated with these advanced delivery vehicles, such as their long-term stability, and introduce the readership to innovative stabilization strategies aimed to extent LNP shelf-life.

## 1. Introduction

Lipid nanoparticles (LNPs) are one of the most clinically tested non-viral delivery systems for nucleic acid-based payloads, particularly for messenger RNA (mRNA). The successful development of RNA-based vaccines during the COVID-19 pandemic has validated LNP formulations as efficient and safe delivery systems. LNPs are currently evaluated in numerous clinical trials for a variety of applications, including infectious diseases, protein replacement therapy, gene therapy and personalized medicine [1,2,3].

**Figure 1:**
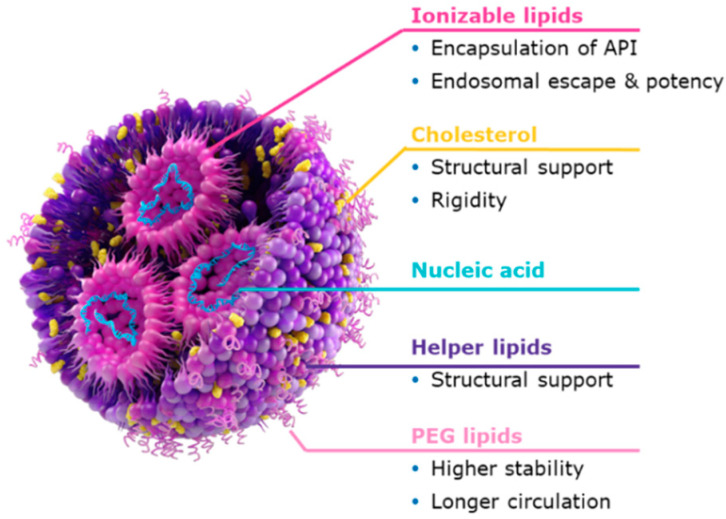
Lipid nanoparticles are the most versatile non-viral delivery system for nucleic acid payloads.

LNPs are particularly complex nanoscale structures that comprise four key lipidic components: an ionizable lipid, a helper lipid, cholesterol and a PEGylated lipid (Figure 1). Each lipid component has a distinct but critical role during particle formation and payload encapsulation, which further affects the biological performance of the delivery system [4,5,6]. The ionizable lipid has two primary roles: firstly it enables the RNA payload encapsulation and secondly it facilitates the endosomal escape at the target site through pH-sensitive protonation [7]. Commonly applied helper lipids, such as DSPC or DOPE, provide structural stability and promote membrane fusion, while cholesterol modulates membrane fluidity and rigidity of the LNPs. The incorporation of PEGylated lipids is crucial for enhancing colloidal stability and prolonging circulation time in the bloodstream [4,5]. LNPs typically encapsulate RNA cargos ranging from ~20 base pairs (bp) to several kilobases (kb), e.g., short payloads such as siRNA and larger ones such as mRNA of 1–5 kb [8]. This reflects the range in which LNPs achieve a balance between efficient encapsulation, nanoparticle stability, and robust protein expression [7].

A commonly described lipid content of the mRNA LNP vaccine is based on the ionizable lipid, a helper lipid, cholesterol, and a PEG lipid at a 50:10:38.5:1.5 mol% ratio, respectively [9]. Though the content of PEG lipid is relatively low, studies by Lokugamage et al. [10] demonstrated that the absence of PEG lipid resulted in large and highly polydisperse LNPs exceeding 200 nm in particle size. Similar studies have shown that including minor PEG lipid content of 0.5 mol% significantly decreases particle size to below 80 nm [9,11]. The underlying mechanism of PEG lipid in colloidal stability is described by the formation of a hydration layer by PEG around the nanoparticles, generating a repulsive force that prevents particle aggregation, further prolonging systemic circulation time. By increasing the PEG lipid content the surface area-to-volume ratio is increased, thus leading to a decrease in LNP particle size [12].

The roles of helper lipids, like DSPC, DOPC, or DOPE, have been tested on the transfection efficiency and stability of self-amplifying RNA (saRNA)-LNP formulations by Barbieri et al. [13] The study reported that DSPC provided the best physicochemical stability profile at high transfection efficiency at the frequently described mol% ratio of 10 mol% helper lipid. A study by Kawaguchi et al. [14] investigated the effect of cholesterol content on the mRNA-LNPs protein expression. The study altered the cholesterol content within the range of 10 mol% to 40 mol%. The result of this study reported a decrease in protein expression at lower cholesterol content, which is further associated with higher degradation in systemic circulating blood after hepatocyte distribution.

The ionizable lipid typically comprises roughly 30–50 mol% of the lipid nanoparticle and directly determines the efficiency of RNA encapsulation, endosomal escape and immunogenicity. A key characteristic of an ionizable lipid is the pKa value, defined as the pH of the ionized and non-ionized form of the lipids at the same concentration. Typical pKa values are between 6 and 7, with studies reporting optimal lipid ranges for intravenous and intramuscular administration at 6.2–6.6 and 6.6–6.9, respectively [15].

Aligned with these studies, the lipid composition, particularly the balance in molar ratios, contributes to the stability, particle integrity, and biological performance of the LNP drug product. Rational LNP design is thus essential as the first step of the discovery phase.

However, developing LNPs remains challenging and requires precise control over formulation composition and process parameters [16]. The manufacturing of LNPs in both academia and industrial perspective starts with the development and optimization of LNP formulations, which includes the critical selection of excipients and the definition of excipient ratios. A systematic approach to accelerate the development and optimization of LNPs is based on Quality by Design (QbD), which is a practical framework commonly employed in industrial development to ensure consistent product quality and compliance.

A practical consideration includes the definition of a quality target product profile (QTPP), which is in alignment with FDA and ICH Q8 (R2) guidelines for early-stage drug development [17]. QTPP is a summary of target quality characteristics of the product and can navigate product and process development, and is highly dependent on the disease target, route of administration, particle composition, payload characteristics, or manufacturing conditions [18,19]. Based on the QTPP, critical quality attributes (CQAs) and their acceptable ranges must be defined. Generally, CQAs include physical, chemical and biological attributes that should be controlled to ensure final drug product quality [20].

In practice, the QTPP of the mRNA-LNP vaccine is defined by a balance of safety (especially for parental administration), high efficacy, and organ-specific delivery. According to that profile, CQAs introduced by EMA for the mRNA-LNP vaccine include particle size, LNP polydispersity, potency (including RNA content, RNA integrity, RNA encapsulation efficiency), and individual lipid content. On the other hand, critical manufacturing attributes (CMAs) include N/P ratio, lipid type, and biodegradability, while critical process parameters (CPPs) include manufacturing conditions (e.g., parameter of microfluidics) [21]. Since CQAs are determined by CMA and CPP, achieving the desired CQAs demands selection of optimal lipids, lipid ratios and manufacturing conditions. Subtle modifications of the components profoundly influence efficacy, safety, and scalability [20,22].

A study demonstrated that altering the N/P ratio (the molar ratio of amine group (N) of the ionizable lipid to that of the phosphate groups (P) of the mRNA) in LNPs can control the payload distribution governed by a kinetically controlled assembly mechanism. Common mRNA-LNPs typically obtain N/P ratios within the range from 3 to 6, with N/P ratio 6 as the most described in studies [4]. Based on optimized and commercially available mRNA LNP vaccines, target specifications of CQAs were defined. As for particle size and PDI values, final LNP drug product should be within the range of 70 to 120 nm and typically <0.3, respectively [23]. A desirable encapsulation efficiency of the mRNA payload is within the range of >80% to >90%, while the zeta potential should be near neutral, to ensure colloidal stability. Concerning the RNA integrity, high percentages of >80% to 90% are favorable [24].

A significant challenge in LNP development is the selection of lipidic components, and the precise molar ratios, as these factors directly dictate the CQAs of the LNPs. Given the central role of the ionizable lipids, LNP development typically prioritizes the identification and screening of novel ionizable lipids. In practice, discovering high-performing formulations in vivo generally requires extensive preclinical screening of large lipid libraries, including hundreds to thousands of lipid candidates [25]. In this early screening phase, fast and robust manufacturing systems are essential to rapidly evaluate large lipid libraries, by minimizing the amount of expensive and limited excipients [26].

Advancements in high-throughput technology and machine learning (ML) are hereby of high interest, especially for the efficient screening of lipids or molar ratios as they provide systematic solutions to the complexity of LNPs [27]. A recent study on the integration of supervised ML in LNP development employs algorithms, such as Support Vector Machines (SVMs), Random Forest (RF) classifiers, Naïve Bayes classifiers, and Multi-Layer Perceptron (MLP) neutral networks, to analyze complex datasets with the aim to predict the performance of LNPs. In particular, large datasets of LNP formulations with varying CMAs, like lipid compositions or N/P ratios, were screened to predict transfection efficiency and post-transfection morphology of the LNP candidates to accelerate the mRNA-LNP therapeutics design for neurodegenerative disorders [28]. Another study by Maharjan et al. [29] optimized the lipid mix ratios of mRNA-LNPs and the microfluidic process conditions using machine learning tools based on Bayesian optimization and self-validated ensemble (SVEM) to optimize the process for efficient LNP preparation.

The rise in Artificial Intelligence (AI) revolutionized the development and manufacturing of mRNA-based therapeutics by further accelerating the optimization of formulation parameters and enhanced production efficiency. Various opportunities exist for AI-driven strategies, ranging from predictive modeling to digital twins, automation, or real-time monitoring, to overcome the limitations of traditional time- and resource-intensive experiments [30].

A recent study by Wang et al., for instance, leverages AI-driven modeling to design ionizable lipids for mRNA-LNP vaccines by predicting the relation between ionizable structure and mRNA delivery efficiency [31]. Meanwhile, other studies employ ML tools or AI-driven models for process automation and control strategy in continuous mRNA vaccine manufacturing by reflecting manufacturing operations through a digital twin.

A more common approach of ML in the LNP manufacturing process employs tools, like analytical technology (PAT), and real-time release testing (RTRT). While PAT provides control over the manufacturing process, RTRT collects information from the manufacturing operations. These combined technologies enable real-time and accurate control of the process. Conclusively, ML tools have improved pharmaceutical manufacturing, firstly by accelerating formulation optimization and secondly by enhancing process control. Subsequently, process control by real-time monitoring, or ML predictive maintenance, has optimized production efficiency and reduced cost [30].

Despite progress in integrated high-throughput and machine learning approaches, the initial step of selecting LNP excipients, such as lipids, often faces several challenges. These include limitations imposed by intellectual property (IP) licenses on the use of various lipids, as well as stringent regulatory requirements related to novel lipids [32]. Selection of helper lipids, further, may be restricted by limited manufacturing, demanding additional costs or generally yielding a high synthesis variability as for PEGylated lipids [33]. The selection of the nucleic acid involves the RNA type, the sequence modification (e.g., pseudouridine or 1-methylpseudouridine), and RNA purity to ensure reduced immunogenicity, high delivery efficiency and high stability [22].

The rapid and adaptable synthesis of RNA, combined with its compatibility for lipid nanoparticle formulations, facilitates swift modifications to address various disease targets. This inherent flexibility positions LNPs as a highly promising delivery system for RNA therapeutics, particularly in the fields of oncology, rare diseases, and regenerative medicine, among others [1,34].

Prior to the clinical manufacturing of an LNP drug product, the formulation design, including optimal excipient selection, ratio optimization and payload encapsulation, must be completed. A key part for the successful development of an LNP drug product includes a robust, scalable, and regulatory-compliant manufacturing process, considering the impact of CPPs on the CQAs of the final product.

This review focuses on the industrial production of nucleic acid-based LNPs, covering the entire manufacturing workflow from upstream unit operations to downstream purification, concentration and final fill and finish. Emphasis is given to process control strategies, including in-process analytical technologies and final product testing. Furthermore, we discuss the key challenges in maintaining LNP stability, both during manufacturing and long-term storage, which today remain critical hurdles for the widespread commercialization of nucleic acid-loaded LNP therapeutics.

## 2. LNP Formation via Nanoprecipitation

LNP formation is commonly achieved via nanoprecipitation, a spontaneous self-assembly process driven by rapid mixing of an aqueous phase containing the payload with a solvent phase of lipids at defined ratios dissolved in a fully water-miscible solvent, like ethanol [35]. Upon mixing, the ethanol phase rapidly diffuses into the aqueous phase, decreasing the solubility of the lipid mixture and inducing their aggregation. The acidic pH of the aqueous buffer (pH 5 or less) protonates the ionizable lipid, making it positively charged. This facilitates electrostatic complexation with the negatively charged nucleic acid, forming a hydrophobic adduct. The hydrophobic nature of this complex leads to precipitation in the mixed solvents, forming hydrophobic nuclei. These nuclei subsequently grow as helper lipids and cholesterol precipitate until a critical point is reached. Finally, PEGylated lipids, which are amphiphilic, absorb onto and penetrate the surface, stabilizing the nanoparticle and preventing further aggregation [36].

Following the mixing step, ethanol concentrations in the formulation usually reach 20–50% (*v*/*v*). While such levels facilitate lipid solubilization and drive self-assembly into LNPs, prolonged exposure can compromise RNA integrity and induce structural rearrangements of the lipid matrix. To prevent these effects, an in-line dilution is typically applied directly downstream of the mixing zone, where a controlled influx of aqueous buffer swiftly reduces the ethanol content to ≤20 vol%. This rapid reduction effectively stabilizes the particle size, limits further lipid reorganization, and protects the encapsulated nucleic acid [37].

Nanoprecipitation is strongly influenced by the mixing attributes, which are governed by two main characteristics, Peclet (Pe) number and Reynolds (Re) number. The Pe number is defined by the relation between the convective transport rate and the diffusive transport rate, which is characterized by fluid velocity and the molecular diffusion coefficient. Pe > 1 reflects slow and less uniform mixing, where mixing is mainly driven by bulk flow rate, rather than the molecular diffusion. Nanoprecipitation at high Pe often leads to broader particle size distribution, with risk of uncontrolled nucleation growth.

In contrast, mixing at Pe < 1 predominantly is characterized by diffusion, where rapid, homogenous mixing at the interfaces yields smaller particles sizes with narrow size distribution [38]. While Pe number described the mixing and transport phenomena in nanoprecipitation, the Re number is a dimensionless quantity that described the fluid flow pattern. The fluid flow regimes thereby are described as laminar (Re < 100) or turbulent (Re > 10,000). Many microfluidic systems in LNP production operate at laminar flow with Re commonly < 100, to ensure stable laminar flow profiles driven by diffusion and controlled interfacial mixing [39]. In practice, mixing devices for LNP manufacturing systems were optimized to operate at laminar flow setups with Pe number < 1 and low Reynolds number for controlled nucleation growth.

The nanoprecipitation process is highly sensitive to the kinetics of mixing, solvent polarity, pH and ionic strength, all of which influence CQAs of LNPs. The formation of stable, homogeneous LNPs depends on precise control over these parameters throughout the mixing process. Efficient LNP assembly thereby requires rapid mixing, typically within a mixing time of milliseconds (optimally in the timescale of 1 to 100 ms) to yield small and narrow distributed particles [40].

The kinetics of mixing dictate the degree of supersaturation and nucleation rate: as an example, by reducing diffusion paths (microfluidics-based mixing), or by inducing chaotic advection and turbulent mixing (T/Y junction or IJM-based mixing), numerous small nuclei with narrow size distributions are produced, whereas poorly controlled hydrodynamics allow uncontrolled growth and broader polydispersity [41,42,43].

Resolving the operational parameters (e.g., Pe and Re number) determined the characteristics of the nanoparticles. Properties, such as particle size and size distribution, are highly related to operational mixing setups. Subsequently, a higher flow rate ratio (FRR) results in a faster dilution kinetic, while an increased total flow rate (TFR) led to higher, uniform nucleation, with both resulting in smaller distributed particles [44].

Traditional manual methods of nanoprecipitation have been complemented by user-independent mixing devices, based on different pumping and mixing approaches. Such devices enable precise mixing of the lipid mixture and RNA in a continuous flow, resulting in highly reproducible mRNA-LNP formulations with controlled size and encapsulation efficiency [45,46,47]. Fine-tuning of FRR and TFR allows robust control over particle polydispersity, while different mixing junction designs enable increased throughput without compromising uniformity, thereby facilitating translation from bench-scale to Good Manufacturing Practice (GMP) production.

Solvent polarity plays a critical role by governing the displacement rate of the organic phase by the aqueous phase; larger polarity differences promote rapid lipid self-assembly into smaller, more uniform particles, while residual ethanol can disrupt interfacial organization and reduce stability if not efficiently removed.

The pH during mixing is particularly important for ionizable lipids, which remain protonated under acidic conditions, enabling strong electrostatic complexation with nucleic acids; subsequent neutralization reduces surface charge, improving biocompatibility and colloidal stability [48]. Elevated ionic strength from salts like sodium chloride reduces the polarity of the aqueous phase, hindering the mixing of ethanol and water, which negatively impacts solvent-exchange kinetics essential for lipid precipitation. This results in less controlled nanoparticle formation, often leading to larger particles with broader size distributions and decreased RNA encapsulation efficiencies [49].

Electrostatic interactions are crucial for RNA loading into LNPs, as the negatively charged RNA binds to positively charged ionizable lipids. Increased salt concentrations weaken these electrostatic forces, leading to lower encapsulation efficiency and unstable LNP structures. Higher salt levels can also promote coalescence and aggregation, compromise colloidal stability and increase particle size and polydispersity indices. At salt concentrations over ~200 mM, LNP stability can collapse, causing visible aggregation. Additionally, salts can slow nanoprecipitation kinetics by altering the diffusion gradient at the ethanol–water interface, disrupting lipid self-assembly.

For optimal LNP quality, nanoprecipitation should occur in low ionic strength buffers to facilitate favorable lipid–RNA interactions. Post-formulation buffer exchange to physiological media should only happen after lipid self-assembly is complete, preserving encapsulation efficiency and particle stability [50].

## 3. From Lab-Scale to Commercial Production

The transition from laboratory-scale LNP production to commercial manufacturing requires a strategic evolution of both formulation processes and process equipment. While small-scale methods, such as manual mixing or benchtop microfluidic devices, are suitable for early research and formulation development, they are often not directly scalable to meet the demands for clinical and commercial supply, particularly under application of GMP conditions.

Thus, key considerations in the industry are focusing on the development of reproducible, scalable, and cost-efficient LNP production platforms to meet industrial quality standards while maintaining the efficacy and safety of the final drug product [4].

**Figure 2:**
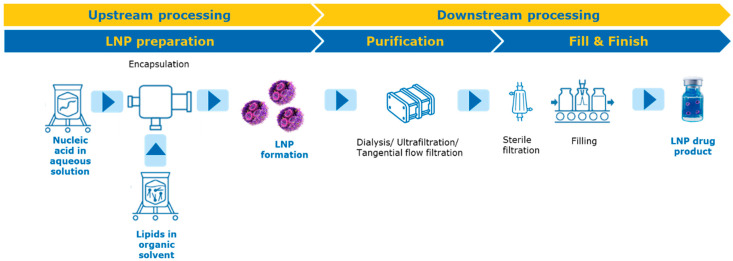
Workflow for LNP production.

Generally, the manufacturing of LNPs follows a two-step approach: (1) upstream processing to form LNPs and (2) downstream processing to remove ethanol, exchange buffer and potentially concentrate (Figure 2). Both processes, upstream and downstream, have to be engineered to accommodate for scale transitions. Accordingly, the choice of equipment, from, e.g., microfluidic chips and syringe pumps to impinging jet mixers and from e.g., dialysis to tangential flow filtration (TFF) systems, plays a central role in enabling reproducibility, scalability, and process control [51].

### 3.1. Upstream Processing

The manufacturing process for LNP therapeutics can be categorized into two mixing types: active and passive mixing systems. Active mixing systems require an external force (e.g., stirring, vibration, sonication, etc.), while passive mixers solely rely on their specific mixing geometry [26]. Both preparation processes can be operated in batch or continuous mode. Since the early 2000s, advancements in continuous processes applying passive mixers to produce nucleic acid-loaded LNPs by ethanol injection have been reported [26,27]. Standard passive micromixers are based on Y-type and T-type junction channels that achieve chaotic mixing conditions through their specific geometries, increasing the contact area and, thus, improving the mixing efficiency [52,53].

#### 3.1.1. Discovery Scale (µL to mL)

Early-stage LNP discovery focuses on rapid, small-scale screening of multiple LNP formulations and parameters to optimize physicochemical properties and delivery efficacy. Approaches, including Design–Make–Test–Analyze (DMTA) or Design of Experiment (DoE) workflows, are employed to identify optimal lipid components and control over CQAs, while working with small quantities of costly materials [18]. These workflows allow rapid identification of promising candidates for further development and scale-up.

Mixer technologies at this stage prioritize flexibility, precision, and speed. In early discovery, microfluidic technology is a preferable choice for LNP manufacturing due to the high reproducibility and the small amount of required materials.

In recent years, syringe-driven microfluidic platforms have gained popularity due to their ability to produce continuous, scalable, and homogenous formulations [16,26]. These platforms employed microfluidic channels to precisely control fluid dynamics by introducing multiple fluid streams into a network of microchannels. The underlying mixing mechanism includes laminar flow, diffusion, and shear forces that support the nanoprecipitation process by rapid mixing within these microchannels. The design of the microchannels can be tailored to optimize mixing efficiency and control the residence time of the fluids.

The most popular microfluidic mixers include T-junction, staggered herringbone (SHM), bifurcated toroidal and hydrodynamic flow focusing (HFF) mixers [54].

In 1999, Hirota et al. first introduced **T-junction mixing** as an alternative preparation method to macroscopic mixing (e.g., pipetting or vortexing) for lipid-based nanoparticles. T-junction mixing provided a controlled mixing environment showing potential towards improved nanoprecipitation [55]. LNP preparation hereby is conducted by turbulent mixing of an organic solvent phase, containing dissolved lipid components and an aqueous solution with the nucleic acid in a T-shaped microchannel.

Common setups employed commercial-grade syringe pumps to inject the phases through the microchannel at very high speeds [56,57]. As the two fluid streams converge at the junction, the high shear forces promote the self-assembly of LNPs. This approach creates uniform and monodisperse nanoparticles, which can be optimized by adjusting critical process parameters (CPPs), including FRR and TFR [58].

Recent studies by Chen et al. [41] systematically investigated an optimized T-junction microreactor on the physicochemical and biological characteristics of LNP formulations. LNPs based on the ionizable lipid, SM102, either loaded with poly(A) or luciferase mRNA were prepared at FFR ranging from 1:1 to 1:7 and TFR from 3 to 36 mL/min in T-junction microreactors. The results demonstrate that increasing TFR reduced the size of the LNPs from 160 nm to 80 nm, with size stabilization at a flow rate above 24 mL/min. Increasing the FFR significantly reduced the particle size and narrow size distribution, with an optimal FRR of 3:1 for poly(A)-loaded LNPs. Under well-established mixing conditions, significantly high payload encapsulation was obtained in that study.

However, a limitation in T-junction mixing is associated with the slow diffusion rate at high TFR, which leads to insufficient mixing, conclusively affects CQAs of LNPs and further leads to batch inconsistency [59].

To accelerate the mixing process, advances in mixing techniques introduced chaotic advection, which manipulates the flow within mixing channels to achieve rapid mixing. This technique employs specific channel designs, such as staggered herringbone mixer (SHM-based cartridge) or bifurcated toroidal mixer [46,60,61]. Currently, **SHM technology** is routinely employed in academia and industrial settings to generate LNPs in a controlled and reproducible manner [62]. Unique microchannel design ensures consistent formulation of LNPs by continuous, circular flow pattern, which enhances the interaction between the lipids and the nucleic acid flow streams. This approach allows more uniform mixing, which minimizes particle size, and polydispersity, which further enhances RNA encapsulation efficiency compared to solely diffusion-based mixing systems (e.g., T-junction mixing).

The quality of LNP formation is mostly determined by key CPPs, including TFR and FRR, between the aqueous and organic phases [43]. A recent study has systematically evaluated the impact of those two parameters on the characteristics of mRNA-LNPs. The study suggested TFRs from 0.1 mL/min to 0.5 mL/min and FRRs from 3:1 to 9:1 as optimal [45]. Another study by Kulkarni et al. with siRNA-loaded LNPs investigated the impact of TFRs within 1 mL/min to 3 mL/min and FRRs within 1:1 to 5:1 on the physicochemical properties of the LNPs [63].

The key observations indicated that the LNP particle size decreases (i) when TFR is held constant and FRR is increased, and (ii) when FRR is kept constant and TFR is increased [45,64]. TFRs in discovery scale of LNP production are within the range of 0.1 to 18 mL/min [46], with 12 mL/min as a commonly applied TFR for microfluidic devices to maintain narrow size distribution and consistent particle size, as demonstrated in the development of Moderna’s RNA-encapsulating LNP vaccines [34]. The underlying principle of particle formation at higher TFRs concludes the rapid supersaturation, which promotes quick particle nucleation and hinders particle growth over time [65].

Similar studies have investigated the impact of FRRs within 1:1 to 6:1 (aqueous to organic) on the physicochemical properties of mRNA-LNPs. The results demonstrated that at an FRR of 3:1 LNP particle size decreased, while RNA encapsulation efficiency was greatly enhanced [46,66]. These findings align with other studies, indicating that a fast dilution of the lipid phase suppresses particle growth, while high encapsulation of the payload is facilitated by rapid lipid assemblies [65,67].

Limitations of diffusion-based microfluidic systems are driven by strict buffer sensitivity, clogging of the microchannel, and restricted scalability. Buffer properties, including buffer viscosity, ionic strength or the presence of salt, directly influence the diffusion rates.

The presence of salts, for example, increases the ionic strength, which affects multiple aspects of LNP formation. Firstly, salts reduce the attractive interaction between positively charged ionizable lipids and negatively charged RNA, resulting in lower encapsulation efficiency due to electrostatic screening. Secondly, high ionic strength can slow the rate of ethanol dilution and alter the diffusion coefficient, leading to larger particle sizes and broader polydispersity. Lastly, elevated salt concentrations can promote premature lipid aggregation and LNP fusion, particularly at physiological salt levels of approximately 150 mM sodium chloride. To mitigate these effects, LNPs are often formulated initially in low-ionic-strength buffers, such as 25 mM citrate (pH ~ 4), and are only transferred into physiological media (e.g., PBS) during downstream purification steps [68].

Further minor limitations associated with this mixing system include environmental waste, high cost due to single-use disposable cartridges, cartridge-to-cartridge variability, and incompatibility of the solvent with the cartridge material [46].

Another microfluidic mixing technique for small-scale LNP production is **hydrodynamic flow focusing (HFF)**. In HFF two fluid streams, a sample fluid and a sheath fluid stream, flow in parallel at different flow rates through a microchannel. The sample fluid stream is thereby focused by the sheath fluid streams to a narrow stream, where mixing at the interface between sample and sheath fluids is facilitated under laminar mixing conditions [57].

In particular, the organic phase (containing the lipid components) flows through the center of a microchannel, whereas the aqueous phase (containing the RNA) is injected from two side-inlet channels at a higher flow rate, to facilitate a compression of the central flow. Common HFF-based devices are integrated with planar 2D geometries, which are less efficient compared to the advanced 3D geometries [69].

The main critical parameters in HFF-based systems include (1) the FRR between the main sample fluid stream and the two sheath fluid streams, (2) the configuration of the microchannel inlet, and (3) the microchannel design itself.

Recent studies by Abdelkarim et al. have investigated different HFF designs (e.g., 2-inlet, 4-inlet) across various sheath inlet angles (45 °C, 90 °C, 135°), and microchannel designs on the physicochemical properties of siRNA-loaded nanoparticles. The results demonstrated that optimal nanoparticle synthesis relies on the interplay of selected flow rate, inlet geometry and the formulation parameter itself [70]. Studies by Jürgens et al. have evaluated lab-scale siRNA and mRNA LNP manufacturing by various microfluidic mixing systems, including HFF on the particle properties and efficiency. The study reported that HFF-based systems allow the preparation of LNPs with small particle size and narrow size distribution with high in vitro and ex vivo efficacy [57].

Despite the rapid preparation of nanoparticles with controlled particle attributes, the limited flow rates of common 2D HFF systems (>10 mL/h) hinder scale-up and high throughput. Even advanced capillary-based 3D HFF systems may have a higher production capacity; however, they are associated with higher operational cost, or risk of dilution of formulations [44,71].

Each microfluidic mixing system offers distinct advantages and limitations, yet they are well suited for the LNP manufacturing at the discovery scale, delivering a fast, reproducible and robust platform for the screening of mRNA-LNP formulations. Moreover, each mixing system provides operational characteristics that are more suitable for scale-up, or operating under GMP standard compared to others (Table 1).

#### 3.1.2. Preclinical Scale (mL–100 s mL)

As the process transitions to medium-scale production, the focus shifts to scalability and reproducibility. In this phase of development, formulation processes must be optimized for later transfer to commercial production, while still being cost-effective and flexible for development. In the preclinical scale, mixing technologies advance towards continuous mixing and parallel processing. Practical establishment in this scale includes pressure-driven microfluidic mixers or mixer chips integrated with peristaltic pumps. These systems are closed, programmable, and allow processing of hundreds of milliliters of formulation under more defined conditions. Mixing systems at this scale provide scalability and GMP compliance to further transfer to commercial scale [51].

Microfluidic-based technologies, initially designed for small-scale applications, can be adapted for medium- to large-scale manufacturing of LNPs through several key strategies. These strategies focus on maintaining control over CQAs while increasing the production rate by transitioning from batch processes to continuous flow operations. Generally, continuous flow operations enable uninterrupted production of LNPs, unlike batch processes that require repeated preparatory steps [72,73].

Another strategy includes the geometric scaling of microchannels. While the small channel size of microfluidic systems offers benefits such as short transfer distances, enlarging the channel dimensions allows higher flow rates for increased throughput without diminishing mixing quality [74].

A more common and effective strategy for scaling up is parallelization or “numbering up”. This involves arranging multiple microfluidic-based channels to operate simultaneously, allowing for the processing of larger volumes of fluids without compromising on precision and controlled LNP production. As presented by Maeki et al. the parallelization of LNP production units, comprising five-layered microchannels, achieves production flow rates within the range of 20–50 mL/min, while maintaining narrow physicochemical properties of the LNPs [73].

Another study by Sheperd et al. presented a scalable, parallelized microfluidic-based system by incorporating an array of 128 mixing channels that operates simultaneously. This strategy achieved a >100 times LNP production rate compared to a single microchannel use, without diminished LNP physicochemical properties and biological potency [59].

Fine-tuning flow rates and mixing conditions in parallelized systems further helps maintain the CQAs of the LNPs.

An alternative to the parallelization of microfluidic-based systems for industrial LNP scale-up production is the employment of impingement jet mixing (IJM) technology. This technology utilizes the collision of two high-velocity liquid streams in a jet mixing chamber, where the aqueous fluid stream mixes with the stream of the organic phase [71]. This process relies on achieving rapid mixing at high Reynolds (Re) numbers (i.e., 100 < Re < 2000). The Re number is an important operational parameter in IJM technology, which is defined by the average velocity at the mixing chamber injectors, the diameter of the injector, and the density and viscosity of the fluid. Mixing thereby is enhanced at high Re numbers resulting in turbulent flow, where inertial forces dominate and facilitate efficient mixing [75].

Rapid mixing and increased shearing forces induced uniform nanoprecipitation that results in narrow and monodisperse LNPs (typically < 100 nm in particle size). This system allows controlled nanoprecipitation by adjusting the critical process parameters such as the pressure and flow rate of the fluids [71].

The use of IJM-based systems in mRNA-loaded LNP vaccine production offers a combination of speed, efficiency, precision and scalability that is not easily achievable with other mixing technologies, making them particularly well-suited for the industrial applications [76].

Due to their robust and scalable LNP preparation, companies routinely utilize IJM-based platforms for large-scale production of LNPs. As reported for the COVID-19 mRNA-LNP vaccine, the scale-up production employed the parallelization of IJMs to meet the high demand of the vaccine [47,48].

Both mixing systems, pressure-driven microfluidic and IJM-based platforms, are suitable for scale-up by generating LNP formulations with desirable physicochemical properties within the specifications. While both can be considered for large scale-up to commercial production, they present different operational challenges, e.g., scale-up of microfluidic-based systems involves parallelized mixing lines to increase the throughput, with the risk of altered mixing regimes of mixer-to-mixer variability. This introduces risks of inconsistent flow rates, fluidic pressure imbalance or channel clogging, often leading to non-uniform LNP product quality [42].

Compared to microfluidic-based parallelized systems, IJM-based platforms generally offer lower complexity while scaling up (Table 2). Thus, they offer the possibility for commercial scale while maintaining homogeneity and controlled CQAs of LNPs [77].

#### 3.1.3. Clinical and Commercial Scale (L–100+L)

Scaling to full GMP-compliant manufacturing requires systems capable of handling liters to hundreds of liters of product, while ensuring sterility and reproducibility. Specifically, the mixing process to prepare RNA-loaded LNPs is among the most difficult operational unit to scale-up to commercial scale, while maintaining controlled CQAs and biological efficacy [78].

A recently introduced commercial formulation system (NanoAssemblr CFS) for commercial-scale LNP manufacturing utilizes NxGen^TM^ mixing technologies enabled rapid scale-up production with operating flow rates up to 800 mL/min (Table 2). The presented study evaluated the quality of a SARS-CoV-2 saRNA-LNP vaccine by the products’ final physicochemical properties and in vitro to in vivo expression. The study could demonstrate reproducible saRNA-LNP production with consistent CQAs from small to commercial scale [78].

Other technologies include large-scale impingement jet mixers, in-line static mixers, and custom-designed continuous flow reactors for scalable, closed-system production under GMP guidelines [79]. These platforms support automation, in-line process monitoring of CPPs, and integration with downstream steps such as TFF and sterile filling lines [72].

The commercial scale production of the SARS-CoV-2 vaccine for instance employed multiple IJM units in parallel with subsequent feeding of the product into a common downstream dilution and diafiltration system to remove ethanol and exchange buffer. Typical flow rates in industrial IJM systems are in the range of 6000–18,000 mL/min per stream, with Re numbers exceeding 10^4^ to ensure turbulent flow [80].

Despite advances in the development of scalable manufacturing devices, or the parallel implementation of mixing systems for commercial-scale LNP production, effective up-scaling remains challenging. Key considerations must be addressed: (1) maintaining the CQAs of LNP, (2) batch-to-batch consistency, (3) sterility and contamination control, (4) adjustment of production technology, (5) highly controlled quality excipients (e.g., lipids), and (6) meeting regulatory requirements.

The most complex hurdles in the scale-up of LNP production is the scalability of the mixing technology, controlled sterility and regulatory demands. Some progress has been made in developing scalable microfluidic systems; however, the capacity for massive volume production remains limited. The practical establishment of sterile and controlled large-scale manufacturing facilities is further associated with high costs and technical demand. Finally, large-scale production facilities must meet the stringent regulatory requirements by authorities like FDA and EMA. Every single step in the production process must be carefully documented and validated to ensure the final products’ quality, safety and efficacy. Conclusively, preparing LNPs at commercial scale for the widespread therapeutic use is still associated with technical complexity and strict regulatory requirements.

### 3.2. Downstream Processing

The downstream processing in LNP production includes purification, buffer exchange, concentration adjustment, sterile filtration and final fill–finish operations to secure a well-defined and consistent quality of the final LNP drug product [81,82].

Particularly, for commercial manufacturing, it is crucial to remove organic solvents (e.g., residual ethanol) from the final drug product to meet regulatory requirements [83]. The regulatory recommendation based on ICH Q3C guidelines for residual solvents, classified as class 3 residual solvents (i.e., less toxic, such as ethanol), should be kept below an exposure limit (EL) of 0.5% in the final LNP drug product.

The presence and timing of salt introduction significantly affect the stability and physicochemical characteristics of LNPs during buffer exchange. Introducing high-ionic-strength buffers (e.g., PBS) too early during the purification process can destabilize LNPs, especially when residual ethanol is still present. Salts such as sodium chloride can create osmotic pressure differences across the dialysis or TFF membrane, which can cause osmotic swelling or shrinkage of LNPs, particularly when lipid bilayers are not yet fully stabilized. Rapid exposure to salts can disrupt electrostatic interactions between ionizable lipids and RNA, leading to RNA leakage, reduced encapsulation efficiency, or aggregation. It is standard practice to perform initial solvent removal in a low-salt or salt-free environment (e.g., citrate buffer or ultrapure water), to minimize coalescence of LNPs due to ionic effects.

Once ethanol is reduced to trace levels (<1%), a gradual buffer exchange to physiological saline (e.g., 1× PBS) is carried out, either via stepwise dialysis or diafiltration using TFF [84]. This stepwise approach helps preserve LNP integrity and ensures optimal physicochemical characteristics.

#### 3.2.1. Small-Scale Downstream Processing

In small-scale downstream processing, standard purification techniques, such as dialysis and ultracentrifugation, are routinely employed.

Despite several disadvantages, such as batch size dependency, potential loss of loaded drug product or generally being a time-consuming method, dialysis is a routinely applied technique due to its high efficiency in buffer exchange, while maintaining sample homogeneity [85,86].

A study by Vargas et al. [86] demonstrated that the dialysis process has a significant impact on the CQAs of LNPs. The study was conducted with unloaded cationic lipid-based (DOTAP) LNP dialyzed against 100 mM KCL in dialysis cassettes with 10,000 molecular weight cutoffs for at least 18 h under continuous stirring at RT. The results demonstrated a significant impact of dialysis on LNPs’ particle size and zeta potential, thus suggesting that dialysis should be considered as a CPP in LNP preparation, accordingly [86].

As an alternative, ultrafiltration using centrifugal filter systems (e.g., with 10–100 kDa molecular weight cutoff (MWCO) membranes) is employed. Ultracentrifugation not only purifies the LNPs but also concentrates on them, which is often necessary for subsequent applications or formulations.

Disadvantages of ultracentrifugation are the potential sample loss and nanoparticle aggregation [87].

Final filtration at this scale is commonly performed with sterile syringe filters, utilizing a 0.22 µm pore size polyethersulfone (PES) membrane. This step is sensitive to particle size and aggregation; therefore, upstream processes must produce LNPs with consistent size distributions well below the filter threshold. Final filling occurs manually under laminar flow in sterile vials. While both methods effectively purify LNP formulations, they are practically restricted to small sample volumes.

#### 3.2.2. Medium- and Large-Scale Downstream Processing

As the process scales up to intermediate volumes, TFF replaces dialysis and ultrafiltration due to its scalability and compatibility with closed-loop systems.

TFF is a filtration technique, also known as crossflow filtration, that primarily is used to purify volumes ranging from a few milliliters to several liters, but can also accommodate much larger volumes, up to thousands of liters. In addition to removing residual solvents, TFF is commonly employed for buffer exchange (often referred to as diafiltration) and up-concentration of formulations.

TFF utilizes porous materials to separate substances based on size [88]. It operates by passing the LNP formulation tangentially across the semi-permeable filter membrane, separating molecules and solvents according to their hydrodynamic size. The pressure applied to the membrane forces small molecules and solvents to pass through the membrane into the permeate stream, while larger species such as LNPs are retained and recirculated back into the feed stream [82,88].

The TFF process can be optimized by considering factors such as membrane material, MWCO, inlet crossflow rate (L/min per m^2^), permeate flux (LMH), product membrane loading (kg/m^2^), transmembrane pressure (psi), surface area, and formulation buffer [81]. Practical advice based on a recent study recommends a low transmembrane pressure (<2.5 psi) to minimize shear-induced stress that might lead to LNP disruption [89]. Meanwhile, the permeate flux should be kept within the range of 10–30 LMH to reduce shear stress and fouling. Recent studies investigate higher permeate flux at >25 LMH in the downstream of LNPs without significant impairment in particle characteristics [90]. In TFF, diafiltration cycles with increasing salt concentrations can be precisely controlled to minimize osmotic shock and prevent aggregation. Membrane selection (e.g., MWCO 100–300 kDa) also affects salt retention and diffusion kinetics; certain salts may accumulate if not properly flushed, potentially altering pH and ionic strength near the LNP surface.

Two common types of TFF membrane modules that are commercially available are hollow fibers and TFF flat sheet cassettes. Flat sheet cassettes are advantageous for processes requiring higher flux and are commonly used in the purification of adeno-associated virus (AVV) products [91]. For biopharmaceutics, including LNPs that are more sensitive to shear forces, TFF systems with hollow fibers are preferred. Hollow fiber modules are cylindrical structures composed of tubular, self-supporting fibers that allow laminar flow, resulting in lower shear forces and gentle processing. Versatile options of membrane chemistry, ranging from more hydrophilic materials like modified polyethersulfone (mPES), which offer an option for higher flux, to more hydrophobic membranes like polyvinylidene fluoride (PVDF) and polysulfone (PS) are available [92]. Filter membranes suitable for the purifying LNPs should not interact with the formulation and ideally have a MWCO of about 100 kDa, as recently described in a patent by Etherna Immunotherapies NV [93].

Despite numerous studies aimed at optimizing and accelerating the process, downstream processing with TFF remains challenging. The typical sizes of lipid nanoparticles (LNPs), ranging from 50 to 120 nm, can lead to increased filtration resistance and operational difficulties, particularly under high pressure [84]. Although the concentrations used in mRNA-LNP vaccines (ranging from 0.5 to 2 mg/mL) do not significantly increase viscosity, they can still lead to other issues during the filtration process. These factors may induce shear forces on the formulation, potentially impacting the stability of the final product [94].

Generally, the purification of LNPs by TFF involves several key challenges, including (1) shear-induced stress that leads to mRNA leakage, (2) membrane fouling and flux decline, (3) membrane incompatibility or (4) lack of process scalability. Particularly, high shear-induced stress at the membrane surface and within the recirculation loop can impair and disrupt the fragile LNP structure, which leads to destabilization of the lipid layer and promoting mRNA leakage. In addition to mRNA leakage, shear can further promote the aggregation of LNPs, which accumulate at the membrane surface and cause membrane fouling. Blocked membrane pores subsequently lead to a significant decline in permeate flux, which results in inconsistent process parameters that impair final filtration quality. As introduced by several studies, the selection of the membrane is crucial to minimize adsorption of the lipid-based product to maintain high recovery of the filtered product. Moving LNP formulations to downstream processes, from laboratory scale to large scale and automated GMP production to the current state, remains challenging in maintaining final LNPs CQAs [84,95].

A key consideration in large-scale downstream processing of commercial-scale LNP production is the transition from manual purification methods, such as dialysis to fully automated cGMP-compliant facilities. Industrial-scale TFF systems handle large volumes efficiently, using single-use components and process automation to perform ethanol removal and buffer exchange with high precision. Sterile filtration is performed through 0.22 µm filters with post-filtration integrity testing, and the final product is aseptically filled using robotic fill–finish systems in cleanroom environments.

## 4. Characterization of Nucleic Acid-Loaded LNPs

The characterization of LNPs is a pivotal factor in the development and commercialization of LNP therapeutics. As the number of nucleic acid-based LNPs entering the market continues to rise, it has become essential to ensure the quality, safety and efficacy of the final product [96].

Bringing novel mRNA-LNP vaccines to the market generally requires comprehensive CMC (chemistry, manufacturing and control) information that reflects the safety and quality consistency of the drug product throughout all manufacturing steps and at every scale. Especially in filing for the approval of the Clinical Trial Application (CTA), this information is required for the IMPD (Investigational Medicinal Product Dossier) in Europe and FDA, and IND (Investigational New Drug) application in the US, as well as EU marketing authorization applications (NDAs) or Biologics License Applications (BLAs). The key objective of CMC is to ensure consistent quality of the LNP product during all phases of development.

The guidelines published by the United States Pharmacopeia (USP) and the European Pharmacopoeia (Ph. Eur.) in 2023 summarize the analytical methods for the characterization and release testing of RNA-loaded LNPs.

**Figure 3:**
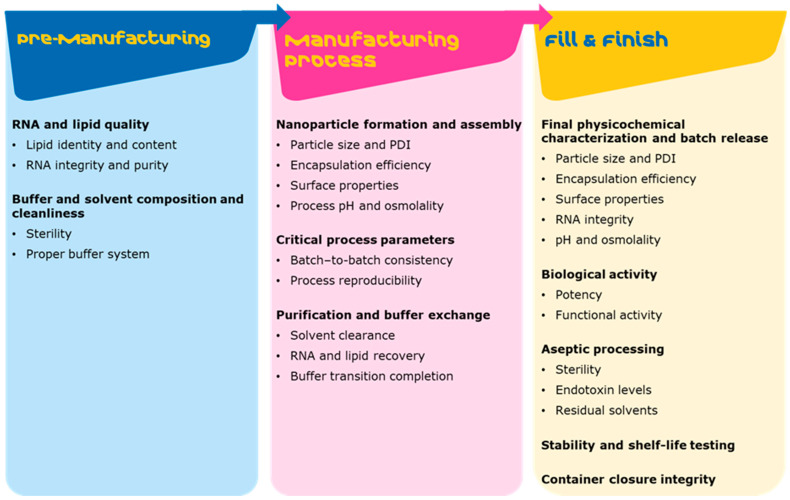
Critical quality attributes of LNPs along the manufacturing process.

The CQAs, defined by the authorities, include physicochemical properties such as (1) particle size and size distribution, (2) payload encapsulation efficiency, (3) surface properties, (4) lipid identity and content, and (5) LNP morphology and biological efficacy and safety assessment of the final LNP product [96,97]. Studies have reported correlations between the physicochemical properties and the efficacy of RNA-LNPs, which further is associated with LNP’s in vivo behavior, including cellular uptake, endosomal escape, biodistribution and immune response [98,99]. Multiple critical process parameters (CPPs) within the LNP manufacturing process, particularly during mixing and downstream processing, will determine the properties of the final drug product, including its safety and efficiency. To maintain the high quality of the final LNP drug product, robust in-process analytics (IPA) and process analytical technology (PAT) are essential to ensure formulation quality and batch-to-batch reproducibility (Figure 3).

### 4.1. Pre-Manufacturing

Prior to the manufacturing of LNPs, a thorough analytical characterization of the excipients, particularly the lipid components and the nucleic acid, is essential to ensure their quality and purity for intended application.

Lipids quality and characterization of impurities are crucial for the successful LNP development. Lipid impurities can significantly impact the stability and effectiveness of the therapeutic cargo, such as mRNA, leading to reduced translation and efficacy. Additionally, these impurities can affect the overall stability of the LNP formulation during storage and handling. To assess the identity and purity of the individual lipidic components, High Performance Liquid Chromatography (HPLC) combined with either a charged aerosol detector (CAD) or an evaporative light scattering detector (ELSD) can be utilized [96]. Valuable techniques in lipid analysis, such as High-Resolution Mass Spectrometry (HR-MS), can analyze the mass of the lipid, while Nuclear Magnetic Resonance (NMR) is used to investigate their molecular structure.

To characterize the nucleic acid-based payload (mainly RNA), well-established analytical methods like gel electrophoresis can be applied to determine the size and integrity. This technique relies on the size-dependent separation of molecules through a gel matrix under the impact of an electric field [100].

A common industrial standard for size analysis is agarose gel electrophoresis, which effectively separates large nucleic acids. Pre-cast agarose gels offer a convenient solution for the rapid and reliable assessment of RNA size and integrity.

A commercially available advanced instrument for nucleic acid characterization is a high-throughput device based on capillary electrophoresis (CE). CE devices provide automated, rapid size and integrity analysis at high resolution within the size range of 10 base pairs (bp) to 40,000 bp [96].

### 4.2. Manufacturing Process

Nanoparticle formation and assembly involve key factors such as particle size and size distribution (PDI), encapsulation efficiency, surface properties, and the process’s pH and osmolality, all of which influence the nanoparticles’ behavior and effectiveness. Critical process parameters, including batch-to-batch consistency and process reproducibility, are essential for ensuring uniformity and reliability in production. Additionally, purification and buffer exchange processes, which encompass solvent clearance, RNA and lipid recovery, and buffer transition completion, are vital for achieving high-quality nanoparticles suitable for biomedical applications. Particle size and size distribution are crucial factors that determine biological efficacy in vitro and in vivo, affecting biodistribution, cellular uptake and immune system activation of nanoparticles. Particle size is classified as a CQA in both preclinical LNP development and GMP-compliant large-scale production [97].

In a typical LNP manufacturing process, particle size and size distribution are among the first parameters measured post-mixing, serving as an in-process analytical parameter that reflects the efficiency of the mixing technology and potential particle aggregation [46]. These parameters are evaluated after critical process steps in both upstream and downstream processes, particularly following mixing (upstream process), buffer exchange via dialysis, concentration adjustment, and sterile filtration (downstream process).

Reproducible LNP formulations, particularly developed for mRNA and siRNA, target a size range within 60 to 120 nm, for optimal cellular uptake and endosomal escape. Meanwhile, a low PDI, typically <0.2 indicating a uniform, monodisperse particle population, is associated with high transfection efficiency.

A common industrial method for analyzing the particle size of LNPs is dynamic light scattering (DLS), which measures the hydrodynamic diameter of nanoparticles within the range of 0.5 to 1000 nm in a colloidal suspension [96,101].

DLS is a fast and simple technology to determine the particle size of LNPs; however, it has several inherent limitations, including low resolution, bias towards larger particles, especially in polydisperse formulations, misinterpretation of spherical particles and sensitivity to aggregates and contaminants [102,103].

Advanced methods, such as multi-angle dynamic light scattering (MADLS), provide a more accurate representation of size distribution in a sample and are routinely used in formulation development and quality control [104].

As nanotherapeutics evolve in complexity, there is an increasing need for sophisticated analytical techniques to characterize the final drug product [97]. Advanced particle size analysis techniques, include asymmetric-flow field-flow fractionation (AF4) and Nanoparticle Tracking Analysis (NTA), offer high resolution particle size analysis, concentration measurements, and structural information [97,105].

Another important CQA is RNA encapsulation efficiency (RNA EE%), which directly correlates with dose accuracy, potency, immunogenicity, and stability of RNA therapeutics [18]. RNA EE% is defined as the percentage of total RNA encapsulated within the LNP, thereby shielding it from enzymatic degradation and premature clearance in the body. This CQA is highly dependent on the formulation, particularly the electrostatic interaction between the cationic charges of the ionizable lipid and the anionic backbone of the RNA. Thus, RNA EE% can be dictated by the selection of ionizable lipid, lipid composition, N/P ratio and the manufacturing parameters [9,106]. RNA EE% is assessed through fluorescent- or absorbance-based assays. In fluorescent-based assays, the interaction of a fluorescent dye (such as Quant-it^TM^ RiboGreen) with the RNA payload is quantified. Another method for determining absolute RNA concentration involves UV-VIS spectroscopy measurement, exploiting the strong absorbance maximum of nucleotides around 260 nm. This method requires disrupting the LNP formulation with detergents like ZWITTERGENT^®^ 3–14 to release the RNA. However, a limitation of this method is the potential interference from other excipients within the LNP formulation that also absorb at 260 nm [96].

Several variables impact encapsulation efficiency, with the selection of lipid components, particularly the ionizable lipid, and the lipid-to -RNA ratio (N/P ratio) being the most notable. Other process-related parameters include mixing geometry and mixing conditions, such as FRR and TFR. RNA leakage may occur during the downstream process, particularly during TFF, due to shear stress.

RNA EE% is measured at critical process steps, including post mixing, after buffer exchange via dialysis or TFF, and after sterile filtration of the final formulation. The specification of RNA EE% typically ranges from 80 to 95%, with commercial production specification defined at ≥90–95% [96].

### 4.3. Fill and Finish

The final characterization of RNA-loaded LNPs is a critical stage in the manufacturing process, as comprehensive datasets are needed to confirm that the drug product meets predefined quality specifications and is suitable for clinical or commercial use.

The surface property of LNPs, commonly described by the zeta potential (ζ-potential), is directly correlated to colloidal stability and the interaction with the cell membrane at the site of action, thus significantly impacting gene expression. The surface charge of LNPs is assessed by analytical assays, such as the TNS assay or zeta potential determination [96].

Parameters such as osmolality and pH are determined to ensure physiological compatibility, particularly when the formulation is intended for parenteral administration. These measurements also support compliance with Ph. Eur. and USP requirements for injectable dosage forms.

The particle morphology of colloidal nanotherapeutics can be analyzed using microscopic techniques, such as transmission electron microscopy (TEM), scanning electron microscopy (SEM), or atomic force microscopy (AFM). Especially cryogenic transmission electron microscopy (Cryo-TEM) has emerged as a powerful characterization tool in the industry, providing distinct morphology data for RNA-LNPs [96,107]. Studies by Szebeni et al. [107] have provided insights into the structure of the COVID-19 vaccine (Comirnaty) using Cryo-TEM. Other state-of-the-art scattering techniques for characterizing complex or colloidal nanoparticles include static light scattering (SLS), small and ultra small angle X-ray scattering (SAXS; USAXS), and neutron scattering (SANS; USANS) [108].

To assess the lipid identity and integrity of each individual lipid in the loaded nanoparticle, conventional chromatographic analytical techniques can be applied. For LNP formulations, the formulation is diluted in an organic solvent to disassemble the nanoparticle, followed by subsequent separation using reversed-phase HPLC-CAD. Recent advancements include the use of IP-RP-HPLC for identifying mRNA impurities resulting from electrophilic degradants and impurities derived from the ionizable lipid [109].

The biological activity, an essential CQA for RNA therapeutics, is assessed through in vitro potency assays that measure translation efficiency or gene silencing, depending on the intended mechanism of action. These assays are typically based on reporter gene expressions in cell cultures or target-specific knockdown quantification via qPCR or ELISA. Functional performance must fall within an established potency range to support product consistency and therapeutic efficacy [110].

To ensure the safety and quality of the final drug product, sterility testing is conducted, typically using membrane filtration methods. Endotoxin levels are determined using the Limulus Amebocyte Lysate (LAL) assay. These tests are crucial for ensuring patient safety and are mandatory for parenteral products. Additionally, residual solvent content (e.g., ethanol from the formulation process) is quantified using gas chromatography (GC/FID) to ensure levels remain below specified toxicological thresholds [111].

Stability testing under real-time, accelerated, and stress conditions is performed in line with ICH guidelines (i.e., ICH Q9) to establish the product’s shelf-life and define appropriate storage conditions, including temperature and light sensitivity [112]. For RNA-based therapeutics, cold chain management is usually required, and freeze–thaw stability is a particularly important factor that must be experimentally verified.

When transitioning from small-scale development to large-scale manufacturing, or when transferring the process to a different production site, comparability studies are performed to demonstrate that the critical quality attributes of the RNA-LNP product remain consistent across sites, batches, and scales. These studies are conducted in accordance with regulatory expectations outlined by agencies such as the FDA and EMA and are essential for supporting regulatory filings, including Investigational New Drug (IND) and Biologics License Application (BLA) submissions.

In summary, final product characterization encompasses a broad array of analytical tests designed to ensure that all CQAs are met in compliance with pharmacopeial and regulatory guidelines. The implementation of validated, GMP-compliant methods throughout this phase forms the analytical backbone of a robust quality control strategy and enables the reliable delivery of safe and effective RNA-LNP therapeutics to patients.

In GMP settings, a complete Certificate of Analysis (COA) for RNA-LNP is standard practice, reporting all relevant parameters (Table 3). Certified reference materials have been developed to support comparability and method validation, including the NRC’s LNP-2, a siRNA-loaded LNP preparation with a reported entrapment efficiency of 94% [113]. In parallel, the U.S. National Institute of Standards and Technology (NIST) is preparing an RNA-LNP Research Grade Test Material (RGTM 10240) to facilitate interlaboratory reproducibility in physicochemical and functional characterization [114]. Moreover, standardization efforts, such as the ASTM international standard for the characterization of RNA in LNP formulations, provide harmonized analytical criteria to support robust COA generation and interpretation [115].

## 5. Current Challenges and Future Perspective for Nucleic Acid-Based LNP Therapeutics

Despite the promising outlook for nucleic acid-based LNP therapeutics, various challenges impeding the progress and widespread application of LNPs are still remaining. One most significant limitation of this novel drug delivery platform is the thermal instability, requiring ultra-cold storage and distribution strategy [116].

During the pandemic, both COVID-19 mRNA-LNP vaccines, Comirnaty^®^ (BioNTech/Pfizer, Mainz, Germany/Kalamazoo, MI, USA) and Spikevax^®^ (Moderna, Cambridge, MA, USA), had to be stored frozen at ultra-low temperatures (e.g., −60 °C to −80 °C), presenting an obstacle to vaccine distribution especially in middle- to low-income countries with poor infrastructure. Advances in terms of long-term storage, especially at elaborate temperatures, cost-efficient transportation and distribution strategies are crucial to meet the future need and applicability of these novel modalities.

Several stabilization strategies are available in the pharmaceutical industry to enhance the stability and integrity of sensitive biopharmaceutical therapeutics. The most common strategies for lipid-based nanoparticles (1) involve the addition of stabilizers to maintain their colloidal stability in aqueous environment or (2) apply lyophilization techniques to increase stability and shelf-life by removing water from the drug product.

### 5.1. Liquid Stability of RNA-Loaded Lipid Nanoparticles

Advancements in the development of stable LNP therapeutics, especially optimal long-term storage conditions in aqueous environments, still remain limited [4]. Several studies could identify potential factors that have an impact on the instability of nucleic acid-based LNP formulations. For example, detailed studies on the commercially available mRNA-LNP COVID-19 vaccines, demonstrated (1) enzymatic degradation, such as hydrolysis and oxidation of mRNA payload, (2) chemical instability of lipid components and (3) the lipid RNA interaction, e.g., oxidative impurities produced by the ionizable lipids, as major factors for the impaired stability of RNA-LNP formulations [116]. On the molecular level, the mRNA payload is fragile and prone to degradation through ribose-mediated backbone cleavage, endo-exonucleases, nucleobase oxidation, and 5′-cap hydrolysis, especially at elevated temperatures. Encapsulating the RNA into LNPs mitigates mRNA degradation; however, it introduces a complex nanostructure system that is associated with chemical instability of the initial lipid components. Degradation thereby arises by lipid oxidation, particularly of unsaturated helper lipids or ionizable lipids, that leads to the formation of reactive species that degrade the mRNA payload [117].

To overcome the instability of RNA-LNPs in aqueous environments, various strategies were employed including modifying the key components of LNPs or developing suitable freezing strategies by adding stabilizers, such as cryoprotectants. Typical cryoprotectants, such as saccharides, amino acids, or polymers, can stabilize colloidal systems by preventing aggregation and maintaining structural integrity during freezing and thawing.

Common applied cryoprotectants in the industry are saccharides. Especially disaccharides, such as sucrose, are known to stabilize LNPs during freezing by directly interacting with polar groups of the lipids to prevent particle fusion. The underlying mechanism is based on the water replacement theory, where saccharides replaced the water molecules that are typically associated with the polar heads of the lipids, thus reducing lipid–water contact [118].

Another stabilization mechanism is described by vitrification. The presence of high saccharide concentrations leads to the formation of a glassy matrix (vitrification) during freezing, which enhances the LNP stability by preventing the formation of physical stress-inducing ice crystals.

In particular, the addition of cryoprotectants increases the glass transition temperature of the freeze-concentrated solution (Tg’), which prevents ice formation during freezing.

Both authorized COVID-19 mRNA-LNP vaccines required sucrose as a cryoprotectant agent for long-term storage at ultra-low temperatures. BioNTech/Pfizer’s (Comirnaty^®^) and Moderna’s (Spikevax^®^) mRNA vaccines are stored in 10 mM Tris buffer containing 10% sucrose (*w*/*v*) and 20 mM Tris buffer containing 8% sucrose (*w*/*v*), respectively. Moderna’s Spikevax^®^ has a shelf-life of 9 months upon storage at −50 °C to −15 °C, while Comirnaty^®^ maintains stability for 12 months upon storage at −90 °C to −60 °C [119]. To date, both vaccines rely on special end-to-end supply cold chain requirements from manufacture to transportation to warehouses and healthcare facilities [120].

Few limited studies have investigated the stability of nucleic acid-loaded LNPs at temperatures other than ultra-low temperatures (like refrigeration, freezing at −20 °C or room temperature). In 2017, studies by Ball et al. investigated the stability of siRNA-loaded LNPs in PBS containing 20% (*w*/*v*) sucrose under various storage conditions, including refrigeration (2 °C to 4 °C), freezing (−20 °C) and room temperature (RT). The findings indicate that LNPs could maintain stability and efficacy when stored at 2 °C to 4 °C for over 150 days, whereas storage at RT led to significant loss of efficacy and physical instability [121,122].

A more recent study explored the effect of different temperatures (e.g., RT, 4 °C, −80 °C) and cryoprotectants, such as sucrose and trehalose, on the stability of nucleic acid-loaded LNPs (e.g., mRNA-LNP, DNA-LNP). Kafetzis et al. [123] reported that 12% sucrose (*w*/*v*) could generally achieve greater levels of payload protection compared to trehalose. Throughout the study it could be demonstrated that particularly mRNA-loaded LNPs were highly sensitive and associated with significant loss in efficacy after 4 weeks upon storage at elevated temperatures (4 °C) or RT [123].

Adding cryoprotectants could improve the stability of LNPs, especially during freezing at ultra-low temperatures of −80 °C to −20 °C; however, sufficient long-term storage at elevated temperatures (>4 °C) remains challenging. Fortunately, advancement in stabilization methods, including freeze-drying, has emerged as a promising technology to obtain dried LNP formulations with enhanced stability for storage and distribution at elevated temperatures [124].

### 5.2. Lyophilization of RNA-Loaded Lipid Nanoparticles

Lyophilization, or freeze-drying, is a well-established technique in the pharmaceutical industry to increase the stability and shelf-life of thermal-sensitive pharmaceutics. In the process of lyophilization, water is removed from the product by sublimation of ice from the frozen state, followed by desorption under a vacuum [125].

The lyophilization process is commonly divided into three stages: freezing, primary drying and secondary drying. In a dried form, lyophilized pharmaceuticals can provide stability without the need of freezing and thus could be distributed worldwide without the need of ultra-cold chain systems [126]. To current date, protein, liposomal or polymeric formulations can be successfully stabilized by lyophilization. However, the lyophilization of nucleic acid-loaded LNPs is not yet fully explored. LNPs are in general highly complex nanostructures, assembled by specific types of lipids at defined ratios. Thus, they are prone to dissemblance due to physical stress and the harsh freeze-drying conditions [127].

The physicochemical instability of LNPs can be characterized as aggregation or fusion of these nanoparticles, which can be determined as an increase in particle size or polydispersity of the formulation. Physical instability can further lead to RNA leakage and thus to degradation of the RNA payload and lipids. The preservation of the LNPs through freeze-drying is coupled to crucial parameters, such as lyophilization buffer, including selection of the cryoprotectant, cycle process parameters and storage temperature.

The selection of lyophilization buffers and cryoprotectants is essential to maintain the stability of LNPs during freezing, which is the first step of the lyophilization process. For the lyophilization of colloidal formulations, several saccharides have been evaluated, including monosaccharides, disaccharides, polysaccharides, and sugar alcohols. Especially non-reducing disaccharides, like sucrose and trehalose, are to date the most investigated cryo- and lyoprotectants in the stabilization of lipid-based nanoparticles by freezing or lyophilization.

Studies by Ball et al. in 2017 have demonstrated that LNP encapsulating small interfering RNA (siRNA) could be successfully lyophilized and stored at −80 °C over a period of 11 months, without significant loss of gene silencing in HeLa cells. The findings of the study indicated that the addition of lyoprotectants, like sucrose and trehalose, up to 20% (*w*/*v*) to the LNP formulation preserved efficacy and maintained siRNA entrapment, narrow particle size and monodispersity of the LNPs [122]. Studies by Muramatsu et al. [128] demonstrated that mRNA-loaded LNP vaccines could be lyophilized and maintain physicochemical properties after storage at RT for 12 weeks and at 4 °C for at least 24 weeks. After respective storage, the study showed that lyophilized firefly luciferase-encoding mRNA-LNPs maintain high expression without decreases in the immunogenicity of influenza virus hemagglutinin-encoding mRNA in comparison to potent mouse immunization.

More recent studies by Ai et al. demonstrated that lyophilized mRNA-LNPs against SARS-CoV-2 were stored at 4 °C and 25 °C up to 6 months without changes in CQAs, such as particle size, polydispersity, lipid content, and mRNA entrapment. The lyophilized formulations could maintain transfection efficiency and elicit potent cellular immunity in mice, rabbits, or rhesus macaques [129].

These studies have demonstrated the feasibility of conventional freeze-drying for the stabilization of nucleic acid-based LNPs. In solid state, LNPs could retain efficacy and stability at specific storage conditions. However, long-term data and comprehensive optimization of the lyophilization process, from the selection of optimal buffer and cryoprotectants to refined freeze-drying process parameters, remain limited.

Beyond conventional lyophilization, alternative drying methods such as continuous vial freeze-drying by spin freezing, spray freeze-drying, thin-film freeze-drying, or vacuum foam drying have been developed and optimized to improve the stability of nucleic acid-based LNP therapeutics [130,131]. Especially, spray freeze-dried RNA-LNPs have gained high interest, since this drying technique primarily provides stabilization of the formulation and further enables the development of pulmonary drug delivery formulations. Other studies employed thin-film freeze-drying to obtain stable powdered solids suitable for oral and nasal administration [131]. To the current state, conventional freeze-drying has shown effectiveness in the long-term stability of the RNA-LNPs vaccine, while alternative drying technologies proved the potential of different administrations of mRNA-LNPs in the solid state. Each drying technology has its own advantages and disadvantages, and most are still being explored.

Conclusively, advancements in drying technologies demonstrate significant potential to overcome the instability of RNA-LNPs, improve shelf-life at elevated temperatures and therefore eliminate the need for ultra-cold chain transport and storage. Embracing AI and ML tools to predict the stability of RNA-LNPs based on the selection of cryoprotectants related to the process parameters or identifying structural changes during the drying processes in correlation to biological efficacy would highly accelerate the development of an efficient drying process.

## 6. Expert Perspective on Transitioning from Preclinical to Clinical Development: Challenges and Solutions

The transition from preclinical to clinical development of nucleic acid-based therapeutics involves several critical steps and common pitfalls that must be navigated to ensure successful outcomes. Establishing CQAs is essential for defining the desired characteristics of the drug product, including purity, potency, and stability. This foundational step guides the development and manufacturing processes, ensuring that the final product meets regulatory requirements and therapeutic efficacy.

As production scales, effective techniques for scaling up manufacturing and implementing tangential flow filtration must consider factors like Reynolds number to optimize fluid dynamics and mixing. The payload-to-surface ratio in TFF systems is crucial for enhancing the purification process while maintaining product integrity. This ratio directly impacts the efficiency of payload encapsulation and overall yield. Therefore, careful management of these parameters is essential during the scaling process. Ultimately, these considerations ensure both the effectiveness and quality of the final product.

A significant challenge in the development of nucleic acid-based lipid nanoparticle medicines is thermal instability, which necessitates ultra-cold storage and presents substantial distribution challenges, particularly in low-resource settings. To address this issue, developing effective stabilization strategies, such as the incorporation of cryoprotectants and the application of lyophilization techniques, is essential for enhancing product stability and facilitating practical storage and distribution solutions.

Several common pitfalls can arise during this transition. For instance, transitioning from research-grade to GMP-grade materials can introduce variability that impacts product consistency. Ensuring consistent quality and availability of raw materials is vital; thus, sourcing from reliable suppliers and conducting thorough quality assessments can mitigate these risks. Additionally, the scalability of analytical methods is critical to align with manufacturing processes. Implementing platform and orthogonal methods, along with establishing predefined system suitability criteria, can enhance the reliability of analytics at scale.

Maintaining consistent particle size and encapsulation efficiency during scale-up presents another challenge. Utilizing Design of Experiments to optimize mixing parameters in clinical mixers and controlling ethanol dilution kinetics are essential strategies to ensure that product quality is maintained throughout the production process. Additionally, it is essential to evaluate filterability and adsorption properties early in the development process. This proactive approach helps identify and mitigate potential problems during sterile filtration, ensuring smoother operations later on. Incorporating pre-filters and conducting thorough validation of filtration processes can significantly enhance the success rates of achieving sterile and high-quality formulations.

By addressing these key aspects, developers can facilitate a smoother transition from preclinical to clinical stages, ultimately enhancing the likelihood of successful product development and regulatory approval.

## Figures and Tables

**Table 1:** Comparison of mixing technologies for LNP manufacturing in discovery phase.

Mixing Technology	Key CQAs (Particle Size and RNA EE%)	Throughput	Scalability	GMP Compliance
T-junction mixer	Size: 50–150 nm RNA EE%: 70–95%	0.1–1 mL/min	- Difficult to scale - Parallelization increases complexity and batch-to-batch variability	- Low to moderate - Requires extensive process validation, closed-system integration, and robust standardization
Staggered herringbone micromixer (SHM)	Size: 40–120 nm RNA EE%: 60–95%	1–10 mL/min	- Scalable by multi-channel scaling - Channel-to-channel variability possible	- Moderate - Single-use or closed-system GMP-complaint devices are commercially available
Hydrodynamic flow focusing (HFF)	Size: 40–100 nm RNA EE%: 60–90%	10–50 mL/min	- High scalability with continuous setups	- High GMP compliance - Continuous manufacturing process with robust control available

**Table 2:** Mixer comparison for LNP manufacturing.

Scale	Mixing Technology	Key Features	Throughput	Key Observations
**Discovery Scale** **(µL to mL)**	T-junction mixer, staggered herringbone micromixer (SHM), bifurcated toroidal, hydrodynamic flow focusing (HFF)	- Rapid screening of LNP formulations - Microfluidic platforms for homogenous formulations - Control over fluid dynamics	0.1–18 mL/min	- Higher flow rates can affect particle uniformity and encapsulation efficiency - Rapid mixing generates steep solubility gradients - Optimal TFR: 12 mL/min for narrow size distribution
**Preclinical Scale** **(mL–100 s mL)**	Pressure-driven microfluidic mixers, impingement jet mixer (IJM)	- Focus on scalability and reproducibility - Continuous mixing and parallel processing - Closed, programmable systems	20–50 mL/min (parallelization)	- Parallelization enhances production capacity - Modular designs support scalability - Challenges with reproducibility in batch-to-batch preparation
**Clinical & Commercial Scale** **(L–100+L)**	Large-scale IJM, in-line static mixers, continuous flow reactors	- GMP-compliant manufacturing - Automation and in-line monitoring - Integration with downstream processes	Up to 700 mL/min per stream	- IJM for SARS-CoV-2 vaccine production - Multiple IJM units operated in parallel - High Re numbers ensure turbulent flow

**Table 3:** Exemplary, non-exhaustive Certificate of Analysis for a LNP drug product.

	Registered Test	Parameter
**Composition**	Appearance	Visual
	Appearance	Visible particles
	Subvisible particular matter	Subvisible particles
	Potentiometry	pH
	Osmometry	Osmolality
	DLS	LNP size, size distribution/polydispersity
	Fluorescence assay	Encapsulation efficiency, RNA content
	HPLC-CAD	Lipid content
	Container content	Extractable volume
**Identity**	HPLC-CAD	Lipid identities
	RT-PCR	Identity of encoded RNA sequence
**Potency**	Cell-based assay	In vitro expression
**Purity**	Capillary gel electrophoresis	RNA integrity
**Adventitious agents**	Endotoxin (LAL)	Bacterial endotoxins
	Sterility	Sterility

## Data Availability

No data was used for the research described in the article.
